# Impact of Integrated Watershed Management on Complex Interlinked Factors Influencing Health: Perceptions of Professional Stakeholders in a Hilly Tribal Area of India

**DOI:** 10.3390/ijerph13030285

**Published:** 2016-03-04

**Authors:** Sandeep S. Nerkar, Ashok J. Tamhankar, Eva Johansson, Cecilia Stålsby Lundborg

**Affiliations:** 1 Department of Public Health Sciences, Global Health—Health Systems and Policy, Karolinska Institutet, Stockholm SE-171 77, Sweden; ejetee@gmail.com (A.J.T.); eva.johansson@ki.se (E.J.); Cecilia.Stalsby.Lundborg@ki.se (C.S.L.); 2 Department of Environmental Medicine, Indian Initiative for Management of Antibiotic Resistance, R.D. Gardi Medical College, Ujjain 456010, India; 3 N.G. Acharya and D.K. Marathe College, Chembur, Mumbai 400071, India

**Keywords:** agriculture, education, healthcare providers, public health, qualitative study, integrated watershed management

## Abstract

Lack of access to water has a significant impact on the health of people in tribal areas, where water in households as well as for productive purposes is essential for life. In resource-limited settings such as hilly tribal areas, implementation of an integrated watershed management programme (IWMP) can have a significant impact on public health by providing a solution to water scarcity and related problems. The professional stakeholders in rural healthcare and development administration are important pillars of the system that implements various programmes and policies of government and non-government organizations, and act as facilitators for the improvement of public health in tribal areas. Information about the perceptions of these stakeholders on public health implications of the integrated watershed management programme is important in this context. A qualitative study was conducted using face to face semi-structured interviews and focus group discussions (FGDs) with stakeholders involved in healthcare provision, education and development administration. The transcripts of interviews and FGDs were analyzed using manifest and latent content analysis. The perceptions and experiences shared by healthcare and development administration stakeholders suggest that implementation of IWMP in tribal areas helps efficient water and agriculture management, which results in improved socio-economic conditions that lead to positive health outcomes.

## 1. Introduction

Fresh water resources are becoming scarce with the increasing demand of the growing population. Globally, 783 million people do not have access to clean drinking water and almost 2.5 billion do not have access to adequate sanitation and this has an impact on their well-being [1]. In India, 144 million people lack access to clean drinking water [2]. Water scarcity combined with inadequate hygiene and sanitation has a massive health impact, particularly from diarrheal diseases [3]. In rural tribal settings, where livelihood is highly dependent on agriculture, water is required for productive purposes such as agricultural activities in addition to household requirements.

In India, indigenous people who live in geographically isolated habitats such as hilly and forest areas, and have distinctive cultures, are recognized as tribes [4]. The tribal population in India is about 104 million and 90% of them live in rural areas [5]. The hilly setting is typical of a tribal area in India and such areas are distributed in several parts of India. The livelihood of these tribal people is dependent on rain-fed agriculture which often suffers from water scarcity [6]. This lack of access to water for productive purposes contributes to a vicious cycle of malnutrition, poverty and ill health [7]. Literature suggests that water scarcity is severe in resource poor settings such as tribal areas, and is responsible for water related diseases such as diarrhea [8,9].

Implementation of an integrated watershed management programme (IWMP) can help solve the problems of water scarcity in an area by increasing water availability and water productivity [10]. Integrated watershed management is a process of formulating and integrating a course of action involving natural and human resources in a watershed, taking into account the social, political, economic and institutional factors operating within the watershed and surrounding river basin and other relevant regions, to achieve specific social objectives [11]. It is the integrated management of land, water and biomass resources within a watershed [12]. An IWMP with community participation and collective action is highly effective in terms of enhanced economic, social and environmental gains [13]. An ideal IWMP focuses not only on soil and water conservation but also on activities like improvement in agriculture, plantation of trees, improving hygiene and sanitation, formation of self-help groups and management of drinking water [14]. The time period required for the implementation of an IWMP varies between different watersheds and may take from four to seven years [14].

An IWMP starts with participation of people by creating awareness regarding water conservation. In the early phase, water conservation works are completed and after achieving water availability, utilization of water for various developmental works is planned and implemented. In the later phase, systematic allocation of resources is done simultaneously creating various dedicated groups to achieve the desired social and economic objectives. An IWMP thus helps in building resilience in rural areas of arid tropics by increasing food production, improvement in livelihoods, generation of social capital and delivery of economic benefits to the population [15]. This study focuses on an IWMP that involves participation of local people. In India, IWMPs that involve participation of people are promoted by the government or NGOs (non-government organizations) or by both in a partnership [16]. All these aspects can have positive impacts on public health.

In our previous qualitative study with villagers in the geographical area same as the study reported in this paper, villagers perceived that IWMP empowered the tribal families in terms of benefits from agriculture, through possibilities for growing new or additional crops, which was seen as empowerment through generation of income as well as employment. Further, self-help groups were mentioned as empowerment for women to help control unsocial things like alcoholism [17]. Quantitative studies, in the same setting, showed a significantly better situation in villages with IWMP as compared to villages without an IWMP, in case of various parameters such as, time period of water scarcity, households with toilet use (56% in integrated watershed management (IWM) villages (IWMV), 15% in Non-IWM Villages (NWMV)), fecal contamination of household drinking water (42% in IWMV, 88% in NWMV), growing fruits or vegetable crops (59% in IWMV, 35% in NWMV) [18]. Further, there were also 2.3 times higher odds for contamination of water sources with various organic and inorganic impurities in case of NWMV [19]. 

Various stakeholders in rural healthcare and development administration, such as healthcare providers, development personnel, education providers, administration staff and non-government organizations (NGOs), are the pillars of the system that implements various schemes and programmes that facilitate development in the tribal areas. These stakeholders monitor the health situation of the people and development in the tribal villages. They are important informers to the policymakers. However, the views of these stakeholders on the relationship between IWMP and public health, have to our knowledge, not been documented. It was therefore of interest to investigate, how stakeholders in tribal areas perceive IWMP in relation to public health. We therefore conducted a qualitative study to explore perceptions of stakeholders of rural healthcare and development administration, on the public health implications of an integrated watershed management programme in a tribal area.

## 2. Experimental Section

### 2.1. Design and Study Setting

**Figure 1:**
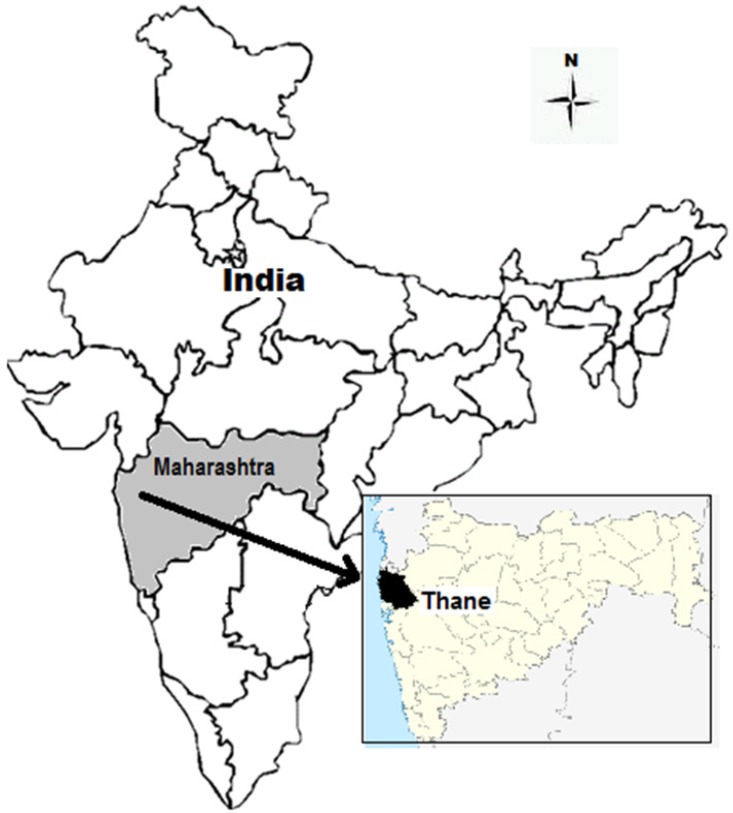
Map of India showing Maharashtra state and Thane district.

An inductive qualitative study using interviews and focus group discussions (FGDs) for data collection and content analysis for analyzing the data was conducted in a tribal area of Thane district in Maharashtra, India (Figure 1). The study area is hilly and the villages, locally referred to as “pada” (which means a hamlet), generally have around 50–60 households. The literacy rate (45%) is far below the national average (74%) [5]. The annual rainfall in the study area (2200–2500 mm) is much above the national average (1100 mm) [20], but as the terrain is hilly, the rain water flows away and the tribal people face scarcity of water during the dry seasons of the year. This suggests a need for conservation and proper management of rain water in the area. Most of the villages only have a tribal population, and all households in these villages have access to common water sources such as open dug wells and rivers [18]. Seasonal agriculture during the rainy season (four months) is the main occupation of the inhabitants, and they often migrate for work during the dry season.

Most health services are provided free of charge by the government through ASHA (Accredited Social Health Activist), primary health centers and community hospitals [21]. There are private physicians in nearby towns, which the villagers may consult. In addition, tribal people frequently seek services from traditional healers called “*Bhagat*”. School education as well as the “mid-day meal” in schools is provided by the government free of charge as a part of national policy. Pre-school (*Anganwadi*) education is provided in each village and a child nutrition scheme is also implemented by the government at the *anganwadi* centers. The various development activities implemented by district and block level administration in the villages are healthcare, education, agriculture, employment schemes and rural development. There is involvement of NGOs in many development schemes such as health, education, agriculture, formation of self-help groups.

### 2.2. Data Collection Procedure

Face to face individual interviews and FGDs were used for data collection. Individual interviews were conducted with stakeholders where group discussions were not possible. These stakeholders were fewer in number and belonged to higher positions in the administrative hierarchical ladder. FGDs were conducted with the stakeholders who were working at village level. In total, 17 individual interviews and four FGDs were conducted. Purposive sampling was used to select the individual participants, for both interviews and FGDs. For the interviews, participants were selected based on the kind of service and experience of work they had in tribal areas, (preferably having experience in the study area for at least one year). Selected participants had work experience in different fields such as health, education, administration and rural development. The details of the participants are described in Table 1 and Table 2.

For the individual interviews, healthcare providers included physicians (4) and drug dispensers (2); development administrators included development officers from government (6) responsible for implementation of various schemes of the government related to rural development, agricultural development, drinking water, women and child nutrition and development officers (2) from NGOs. Education providers were teachers. Two development administrators were female; all the other interviewees were male. We followed an emergent design to saturate the data and to decide on the number of interviews and FGDs needed. The number of FGDs and individual interviews were thus not decided beforehand but was concluded when saturation was reached, *i.e.*, when no new information came up. 

We tried to maintain homogeneity within each FGD with regard to sex and education. A guide was prepared with open ended introductory questions for the areas to be explored (Table 3).

The same guide was used both for individual interviews and FGDs. The guide was piloted before use. The first author (SSN) was the moderator for all the FGDs, while an additional female moderator was included for the FGD with women. The moderators of the FGDs had a good understanding of the customs and traditions of the study area. All interviews and FGDs were conducted in the local language “Marathi” and the discussions lasted between 45 to 90 min. The number of participants in each FGD was between seven and nine. The FGDs were conducted at places and times convenient for participants as well as moderators. All interviews were conducted by the first author (SSN), recorded using a digital voice recorder, and an observer was present for note taking. In seven cases, where recording was not agreeable to the interviewees, running notes were taken by the interviewer and a note-taker separately.

### 2.3. Data Management and Analysis

The recorded interviews and FGDs were transcribed verbatim in Marathi. The transcripts in Marathi were used for identifying codes to understand the actual meaning of the text. These codes, along with relevant quotations, were translated into English by the first author (SSN). These translations were cross-checked by co-author (AJT), having good command of both languages. Manifest and latent content analysis were used as methods for analysis. No pre-determined themes or categories were used. Manifest content analysis is an analysis of what the text directly says and thus describes “the visible”, while latent content analysis concerns an interpretation of the underlying meaning of the text [22]. First, open codes were identified in the text. These codes were grouped into sub-categories according to similar patterns. The sub-categories were further grouped to form categories to achieve the manifest level of the content analysis. During the analysis, all codes and categories were cross-checked by all authors. Finally, the underlying meaning of the text was identified as a theme.

The first and second authors have a good understanding and long experience in participating in projects in rural India. All authors have a public health perspective and experience with qualitative research, and have varied professional backgrounds viz. agriculture, environmental health, nursing and pharmacy. The different nationalities, genders, expertise and experiences of the authors brought their unique perspectives to the analysis of the study.

### 2.4. Ethical Considerations

This study was approved by the Institutional Ethics Committee of the R.D. Gardi Medical College, Ujjain, India (No. 175/2011). Informed verbal consent was obtained from all participants. Participation was voluntary and the participants were informed that they could withdraw at any time from the study without any implications. To maintain the confidentiality of the interviewees, we have used broad terms in identifying quotations from different stakeholders.

## 3. Results

One theme, “Possible impact of integrated watershed management on complex interlinked factors influencing health of tribal people” was identified from the analysis (Table 4). Six categories were described in the manifest content analysis (i) impact of water scarcity on health issues; (ii) effects of lack of education on health and utilization of healthcare facilities; (iii) role of traditions on women’s and children’s health; (iv) dependence of economy on agriculture; (v) efforts of the government to provide/improve education, employment and healthcare and (vi) possible impact of watershed management on economy, employment and education. Relevant quotes from the interviews and FGDs are presented under the description of each category to illustrate the findings. If necessary, an explanation of the context is given in square bracket within quotes.

While presenting the findings of the study, we first explain the problems of tribal people as perceived by the participants and then present their views on the possible impact of IWMP on various health problems of tribal people.

### 3.1. Impact of Water Scarcity on Health Issues

All the participants perceived that in general, there was water scarcity in the area where the tribal villages were located, and observed that water scarcity occurs in non-rainy season, with increased severity during the summer months. According to the participants, tribal people usually fetch water from nearby water sources, mostly wells as no piped water is available. During the summer months, the sources become dry, forcing people to go to more distant sources. Participants also stated that there is surplus rainfall in rainy season, but the villages are located on top of hills and water flows away due to the slopes. In the majority of villages there is no conservation of water and there is a lack of management of the water supply.

“*In this area, most of the villages are declared as water scarce (in summer)……from March to May water scarcity usually becomes severe*” (Development officer, 30 years’ experience).

While sharing their experiences of the impact on IWMP on water scarcity, participants perceived that there was an increase in water availability in villages where IWMP was implemented.

“*Earlier in that village (where the integrated watershed management programme was implemented), there was water scarcity. Now there are trees and every nala (stream) has water flowing*” (FGD, Local leaders).

Participants perceived that the hygiene and sanitation practices followed by tribal people were improper, and water scarcity was one of the reasons responsible for this. Open air defecation was a common practice in the area. Participants stated that water scarcity is one of the major reasons for open-air defecation and the subsequent contamination of water in community water wells.

“*We say, that every house should build a toilet…but an important problem is, that they (tribal people) do not have water to drink, ……so how could they have water for toilet use. That is why they go to open air places (for defecation).*” (Teacher, 15 years’ experience).

(Even for dry toilets about 1–2 liters of water is required, because in India it is customary to use water instead of toilet paper for cleaning up after defecation).

According to the participants, stomach problems and diarrheal diseases were very common illnesses in the area. Other illnesses included fever, colds, skin infections, eye infections, hepatitis, pneumonia and malaria. Participants felt that all these illnesses were related to water scarcity, resulting from improper hygiene practices such as irregular bathing, hand washing negligence and insufficient care of infants.

“*In these villages, diarrheal diseases are most prevalent, ……there is contamination of water, lack of hygiene (at household level),….fever and cold with stomach problems.*” (FGD, Frontline healthcare providers).

However, participants perceived that in the integrated watershed management villages, hygiene and sanitation was better compared to other villages.

“*In the integrated watershed management village, cleanliness is there, water source is located close, facilitating taking baths and washing of clothes.*” (FGD, Frontline healthcare providers).

All the participants perceived that tribal people grow food-grain crops only in the rainy season, and that is mainly for household use. There is enough stock of food-grains like rice and *nagali* in tribal houses. Vegetable consumption is very low as they do not have water to cultivate crops in non-rainy season and have no purchasing capacity. According to participants, tribal people neither get enough calories nor a balanced diet, and malnutrition is a serious health issue in the area.

### 3.2. Effects of Lack of Education on Health and Utilization of Healthcare Facilities

All participants stated that there is a lack of education among tribal people. Moreover, the participants perceived education as highly neglected in the tribal communities as the villagers did not understand the importance of education. According to the participants, due to high health illiteracy, tribal people do not follow improved hygiene and sanitation practices, which adversely affected their health. Participants explained this by quoting a few examples such as dislike for chlorination of water and misconception regarding family planning and use of contraceptives. Lack of education was also perceived to have an effect on the utilization of formal healthcare services, even though these services were provided free of charge by the government.

*“(General and health) illiteracy is a big problem in this area, ……even someone who becomes a (political) leader, he does not use the toilet; ……for this, (lack of) education is responsible……When there is illness, instead of going to the healthcare center, these people get exploited by superstitions….there is a bhagat (traditional healer) system, people go there, whatever may be the severity of illness.*” (FGD, Teachers).

Participants perceived that lack of education is one of the most important reasons for excessive consumption of alcohol in men, and malnutrition in the family. Participants perceived that education could reduce the problem of alcohol addiction in men, which is very common in the tribal community and affects the whole family.

“*Some parents (mainly men), who are illiterate, spend all money which they have got from the scheme (for the nutrition of pregnant women) on alcohol; their children are malnourished.*” (FGD, Teachers).

Participants perceived that the people from IWMP implemented villages are more aware of importance of education of their children, and were more positive in utilizing various services provided by the government. Participants viewed that this happened because of the development in their villages.

“*Among all villages, the integrated watershed management village is developed, we can see trees there… people focus on their work, plant wadis (small fruit orchards), educate their children……and are also responsive (to various schemes of the government).*” (FGD, Frontline healthcare providers).

### 3.3. Role of Traditions on Women’s and Children’s Health

Participants perceived that tribal people consume very small amounts of milk, oil and leafy vegetables, and therefore lack essential nutrients. According to the participants, many traditions exist in tribal population that have an adverse impact on their health, such as an early marriage system, typical neonatal care, traditional healthcare, division of labor on gender basis and alcohol use during festival times. These have effects on tribal people’s health. Participants perceived that tribal people had knowledge of herbal medicine, but they did not put much emphasis on it. Moreover, participants perceived that tribal people linked this knowledge to superstitious practices and to their traditions.

“*Tribal people know many medicinal plants that come in forest, which can work better on normal fever, cough and even injuries or fractures, ……they know one herb that works very well on kidney stone.*” (Drug dispenser, 8 years’ experience).

Participants viewed that traditionally women have to bear the responsibility for all household work. This includes collection of water and firewood, cooking, maintaining the cleanliness of the household, taking care of the children and farm labor. According to participants, all this hard work continues even during pregnancy and was perceived to affect women’s health. Lack of proper food resulted in underweight children. Participants perceived that traditionally women and girls have to bear the responsibility of fetching water from distant sources and water scarcity adversely affected women’s health.

“*In one village, there was a camp on Hb (haemoglobin) testing. Out of 15–20, there were seven women having low Hb……means blood is less in quantity, but she does all the work*.” (FGD, Local leaders).

The heavy workload at home and at the farm suppresses women. Women do not get enough nutrition, which results in anemia, especially in water scarce areas. One of the participants shared his experience as follows.

“*We were working in nine (tribal) blocks, organizing health camps,……we observed that the Hb of women from one particular block was very low……and that the same block faces severe water scarcity*.” (Development officer, 10 years’ experience).

All participants stated that there is a tradition of early age marriages in the tribal communities, which adversely affects both women’s and children’s health. According to them, girls get married in adolescence and therefore their education cannot be continued. This also creates health problems like malnutrition in children and anemia in women.

“*Parents arrange the marriage of their daughter at the age of 15–16 years. Some girls become pregnant even at 14 years. They don’t know what pregnancy is. In that case operation (abortion) or delivery is not safe. Many girls don’t know how they became pregnant, no education at all……in this area early age marriages is a serious concern. People need education and knowledge. They should know more about what is going on in the rest of the world*.” (Physician, 10 years’ experience).

Participants stated that there is a tradition of ceremonial bathing of neonates in the open air with ambient temperature water. According to participants, there is a traditional misbelief among tribal communities that bathing of neonates in the open will develop their immune system. Participants also perceived that this practice leads to pneumonia in children and is a cause of infant mortality, which is a major public health concern in the area.

“*Immediately after birth, by detaching the umbilical cord, the newly born infant is put into normal water for bath. According to the tribal people it makes baby strong*.” (Teacher, 15 years’ experience).

### 3.4. Dependence of Economy on Agriculture

All the participants perceived that the economy in the tribal area is totally dependent on agriculture and that most of the tribal people have farming as their main occupation. Participants viewed that farming is dependent on monsoon rain and in other seasons there is no work in the tribal villages. Unemployment in non-rainy season forces the tribal people to migrate to other places to earn money.

“*In this area, crops are cultivated only in the rainy season; in the summer season there is a problem even for drinking water, how could farming be possible*.” (FGD, Frontline healthcare providers).

Participants also perceived that although agriculture is the main occupation of the tribal people, it is underdeveloped, and tribal people lacked sufficient knowledge of how to cultivate different crops. According to participants, tribal people mostly grow cereal crops. Rice and a millet crop, *Nagali,* are meant for self-consumption. *Bhagar*, a millet crop, is the only cereal crop grown for sale, but without much monetary gain. Participants perceived that tribal people do not know the importance of irrigation and lack willingness to develop the agriculture by proper utilization of water. At the same time, participants also perceived that in a few villages, people took initiative and developed their agriculture by effective utilization of water. The farmers got good economic gain from their fruit plant produce and vegetable and flower cultivation. Participants perceived that earnings from more developed agriculture reduced migration in some villages. They also perceived that there should be a change in the cropping pattern to bring change in the economy of tribal people.

“*In this area, people should take a crop like groundnut. That can generate income, make enough edible oil available and also its by-products would be used as excellent cattle feed.*” (Development officer, 35 years’ experience).

### 3.5. Efforts of the Government to Provide/Improve Education, Employment and Healthcare

Participants perceived that the government made all possible efforts to improve education, to provide employment and to increase the utilization of modern healthcare services in the tribal areas. Most of the participants admired the government’s programme on education and nutrition in pre-school and school through *anganwadi* workers and teachers, which had shown a direct impact on the health of tribal children. According to the participants, *anganwadi* workers are more attentive to nutrition of the children than their parents. Participants felt helpless that children discontinued their education because of socio-economic reasons, and due to this, the effectiveness of good government programmes was lost. Participants perceived migration as a major obstacle to the implementation of various governmental schemes. Seasonal migration affected tribal people with respect to education of children, addiction to alcohol and many health problems. Addiction to alcohol was perceived to become severe when men migrated without their families.

“*The government provides education facilities (to tribal people) through various schemes, but the economic condition of the people is not good, ……people have problems with food, so what can they do with education……they remain away from education……Migration affects the attendance (of the student) in school……from December to January 50% of the students are out of the village*.” (FGD, Teachers).

Participants perceived that migration from the IWMV had reduced due to the generation of employment in agriculture in those villages. This also reduced the extra burden of work on women, and suffering of families due to migration. Participants viewed that migration was reduced to a certain extent in IWM villages.

“*Earlier it was the same (in IWMV) as in other villages (non watershed management villages), people were migrating to other places for employment, but nowadays in integrated watershed management village not many people go. Women remain in villages, only a few men go for sand work, but earlier the entire family was migrating*.” (FGD, Frontline healthcare providers).

Participants stated that there are various governmental schemes that are meant to generate employment opportunities in the village/area. These schemes however do not attract tribal people, they migrate to cities or other areas mainly to do brick work or sand work. The local community leaders in the FGDs perceived that there were a number of government schemes which the local tribal people were not aware of due to illiteracy.

Participants perceived that healthcare services provided by the government mainly focused on curative measures and less on preventive measures. Participants perceived that taking proper preventive measures could solve many health problems in the tribal community, and thought that there is a lack of co-ordination between different departments and agencies working for the same cause.

In summary, though there are many schemes planned and implemented by the government, participants perceived that there is lack of coordination between various departments and therefore effectiveness of the programmes is not seen.

### 3.6. Possible Impact of Integrated Watershed Management on Economy, Employment and Education

All participants perceived the need for water availability in all seasons in the area, and emphasized the need for conservation of rainfall water and its effective utilization. IWMP was viewed as helpful in the conservation of water as well as for improvements in agriculture.

“*If an integrated watershed management programme is implemented effectively, water storage will be utilized for agriculture; if they get income from agriculture, their livelihood will improve, and economic condition will improve and education, health will also improve. ……Improvement in education and health will come only after economic development*.” (FGD, Teachers).

Participants also shared their experiences about the impact of IWMP in villages.

“*In the integrated watershed management village, programmes like wadi (a small fruit orchard) were provided, (documentary) films were shown, ……they (implementing agency) told about self-help groups, a short story of alcohol addicted men……also performed drama (to create awareness)*.” (FGD, Frontline healthcare providers).

“*We completed the drinking water schemes through the integrated watershed management project……earlier people were facing a lot of trouble to get drinking water; they had to go for long distances. People (from other villages) were not ready to marry their daughters to young men living in the (water scarce) village because that village was on high elevation and people there (mainly women) had to go down and bring 2–3 big pots of water on their heads. Now (after implementation of IWMP) almost 90% of the young men got married (because of water availability).*” (Development officer, 10 years’ experience).

Participants also perceived that there was a need to improve the nutrition of the tribal people. Development in agriculture and improvement in economic condition of the tribal people might be the solution to the problem of nutrition. Participants also perceived that according to physical appearances, the people from IWMV were healthier than people from other villages.

“*If there is water availability, some people will cultivate vegetables ……it will generate some kind of employment in the village itself……if there are vegetables growing in the villages, then vegetable consumption (by villagers) will definitely increase.*” (FGD, Local leaders)

Generation of employment and increase in income at the village level was perceived as the most important factor in reducing alcoholism in men. In the village, there is social accountability of individuals to their families and money earned will not be easily spent on alcohol. Furthermore, participants stated the possibility of generation of employment by plantation of fruit crops through the implementation of IWMP, which could help to reduce migration and the continuation of education of children.

“*Generation of employment will increase the income and then everyone will think of their children’s education*.” (FGD, Local leaders).

Participants perceived that an integrated approach is good to improve socio-economic conditions, and effective implementation of such programmes is important but it takes time to see its effect.

## 4. Discussion

The perceptions and experiences put forward in this study suggest that health problems of tribal communities are multifaceted and interlinked to determinants of health such as economy and education. According to participants, the economy in the study area is dependent on agriculture, which is basically under-developed, and water availability is the main hurdle in its development. The views of participants suggest that an improvement in agriculture may result in an improvement in nutrition, economy and generation of employment. These improvements further lead to improvements in education and awareness among tribal communities, which ultimately will have an impact on their health. Overall, the perceptions and experiences shared by professional stakeholders suggest that implementation of an integrated watershed management programme in a tribal area helps in increasing water availability and improvement in agriculture. This will consequently have a positive impact on economy, education and ultimately on public health.

### 4.1. Impact of Water Scarcity on Health Issues

Participants perceived that water scarcity affects many hygiene and sanitation practices of the tribal people in the study area, and causes them to suffer from various water related diseases, such as diarrheal diseases. These findings are consistent with the findings of a study from Nigeria, which suggests that diarrheal diseases such as cholera, typhoid fever, salmonellosis, gastro-intestinal illnesses and dysentery were the health consequences of water scarcity [7]. A study from Zimbabwe also stated that water scarcity was responsible for water-related diseases and environmental contamination in their settings and also suggested that water scarcity is more severe in resource poor settings [8]. This supports our findings regarding consequences of water scarcity for tribal people. In the study area, participants perceived that the occurrence of skin infections and gastro-intestinal disorders (stomach problems) were due to water scarcity. In a study from rural Alaska also, natives suggested that the risk of skin and gastro-intestinal infections increases, when there is no home-water service [23], which is in line with our findings. In the study area, although there was no water supply system directly to houses, participants perceived that people from IWM villages had water sources closer to their houses, which helped them to maintain hygiene at the household level better than in non-watershed management (NWM) villages. Our earlier study from the same tribal region showed that during the scarcity period, people from IWMP implemented villages had water sources closer to their houses compared to villages where it was not implemented. Further there were several significant differences in hygiene and sanitation practices between the two settings, some of which are mentioned in the introduction (like availability of toilets in the household in IWMV, which reduced open air defecation practices) [18]. 

### 4.2. Effects of Lack of Education on Health and Utilization of Healthcare Facilities

Participants perceived that many health problems of tribal people are indirectly associated with lack of education. For example, lack of knowledge about proper hygiene and sanitation lead to infectious diseases. It also, leads to misconceptions regarding health interventions proposed by the government, and this poses a challenge to the government to propagate new technologies among tribal people. It is a known fact that tribal people are lagging behind the general population in many parts of the world with regards to various developmental aspects including education [24]. To improve the public health situation in a tribal area, there should be increased utilization of improved healthcare facilities that can be facilitated by educating the people about their health. A study from Peru suggests that formal education imparted to women influences the use of maternal healthcare services [25]. Education of women may be helpful in improving maternal and child health in indigenous or tribal societies living in resource poor settings. In the study area, participants emphasized the need for girls’ and women’s education and felt that water scarcity sometimes becomes a hurdle for continuation of education of girls as they are responsible for fetching of water. Participants also viewed that an improvement in water availability and agriculture reduces migration. This helps to retain girls as well as boys in school education. This perception is supported by our earlier quantitative study from the same tribal region which showed that greater number of girls (odds ratio = 3.4) were able to continue their education in villages where IWMP was implemented (80 of 90 in IWMV compared to 63 of 87 in NWMV (*p* < 0.005) [18]. 

Alcohol addiction in men in tribal societies was perceived as a common phenomenon and lack of education was perceived as one of the reasons for excessive consumption of alcohol, which affected overall family health. This is documented in another study from rural India, which stated that men with no formal education in scheduled tribes or castes consume high amounts of alcohol [26]. Increasing awareness about education, generation of employment and reduction in migration due to implementation of IWMP might be helpful in the reduction of excess alcohol consumption in men and health problems associated with it. Similar views were perceived by villagers in our earlier study [17].

Participants perceived that tribal people have knowledge of local herbs that have medicinal properties, but tribal people gave little importance to their knowledge. It is believed that tribal people have the knowledge of herbs that are being used as traditional medicine, and there is a need for conservation of both, the plants as well as the associated knowledge [27,28]. It is already a possibility that in the study area, there might have been a loss of medicinal plant species with increasing deforestation. Traditional knowledge of medicinal herbs is also losing its importance as it is entangled with superstitious practices.

### 4.3. Role of Traditions on Women’s and Children’s Health

The practice of marriages at an early age in the study area was perceived by participants as a reason for many health problems of women and children. There are studies from Africa and India reporting the existence of early age marriages in tribal societies and the associated health problems in women and children [29,30]. Findings of our study are consistent with these studies and suggest that there is a need to educate and to create awareness among tribal people to change their attitude to early age marriages and empower women. In our earlier study, tribal villagers perceived IWMP as helpful in empowering women and girls by generating social capital and facilitating girls’ education [17].

Participants stated that there are many traditions that exist in tribal communities that adversely affect their health. Bathing neonates in an open environment is a good example, as it is perceived to result in pneumonia. Similar types of traditional new born care practices have earlier been reported from India [31,32]. Educating people about correct practices is very important in tribal areas where infant mortality is a major public health concern.

### 4.4. Dependence of Economy on Agriculture

Participants perceived that tribal people in the study area did not receive sufficient nutrients and calories from their food, as their diet mainly came from agriculture that is under-developed and dependent on rain. According to the participants, malnutrition is a serious concern in the study area, for which lack of water for agriculture and hygiene and sanitation, is the main cause. Malnutrition in the study area was also reported to be associated with an increased under-five mortality rate [33]. According to the participants, malnutrition in the study area is a result of multi-factorial processes. The perceptions of study participants are consistent with a study which states that areas where a major portion of food is derived from primitive agriculture, it results in under-nutrition in children [34]. This suggests that in such areas, improvement in agriculture with efficient use of water is very important to overcome the problems of malnutrition. 

There are studies that show that watershed management increases the water availability and that this can be a solution for water scarcity, especially in the tropics, where agriculture is dependent on it [10,35]. Development of agriculture becomes is important as most of the food is derived from it. In our earlier study, significant improvements in agriculture and production of fruits and vegetables were noticed in IWMP implemented villages compared to villages where it was not implemented. Furthermore, purchased fruits and vegetables were consumed to a significantly higher extent in IWMP villages [18].

### 4.5. Efforts of the Government to Provide/Improve Education, Employment and Healthcare

There are various schemes formed by the government for the development of the tribal people. According to the participants, many schemes do not achieve the success that is expected. There are studies on employment and nutrition schemes in India that mention similar views on the implementation of these schemes [36,37]. However, healthcare services provided by the government through female workers were perceived as successful in the study area. Participants mentioned the importance of the role of *anganwadi* workers in supplementary nutritional programmes as well as the role of ASHA on promoting institutional birth deliveries and utilization of healthcare services. There are reports that support these findings which inform the success of national rural health mission (NRHM) of India in delivering services through ASHA [38]. 

As far as the healthcare sector is concerned, participants perceived that government schemes focused more on curative measures than preventive measures. This was also true with the healthcare approach for water related diseases in other parts of the world including Ghana and Mexico [39,40,41].

Participants perceived that there was an increase in water availability in IWM villages and water was used for the development of agriculture in those villages. This perception is also supported by the findings from our earlier study, which reported a significantly lower percentage (14% *vs.* 88%; *p* < 0.001) of households from IWMV experiencing prolonged water scarcity compared to NWMV [18].

### 4.6. Possible Impact of Integrated Watershed Management on Economy, Employment and Education

In general, participants perceived that the income of the tribal families is meagre and unemployment during non-rainy season forces them to migrate to other places for earning their livelihood. Migration in the study area was viewed as a hurdle in the education of children. There is a well-established positive association between health and education [42]. Participants emphasized that a low level of health awareness due to low level of literacy is the underlying cause of many health problems and it is very important to improve the situation regarding education and awareness to improve the public health in the study area. Employment generation at a local level through development of agriculture was perceived as important in improving the economic conditions of households and for education of children. This suggests that the implementation of IWMP can help to improve education and economy, and therefore is positively associated with improved public health.

### 4.7. Methodological Considerations

In this study, we conducted both FGDs and individual interviews. FGDs were conducted with stakeholders, who were at the base of the hierarchical ladder of administration and there we maintained as much homogeneity as possible within each FGD group to facilitate an open discussion. For other stakeholders individual interviews were considered as a better alternative both to elicit the views of the participants and also for feasibility reasons. The stakeholders higher up in the hierarchy were few and scattered over large geographical area and also had specific education and tasks, thus individual interviews were a better alternative. The same guide was used for both individual interviews and FGDs so as to explore the areas of interest from a range of perspectives. This is a qualitative study, thus we use the term transferability and not generalizability, and the results cannot be directly generalized to other groups or settings, but may be transferred. The number of interviews or FGDs was not decided beforehand, but data were continued until data saturation was achieved, *i.e.*, no new information came up. We can of course not exclude that other individuals might have had other views, however the views we elicited were present among the participants and are as such important to present.

During the data analysis, cross checking of the transcripts and translations, with recorded data was performed to avoid misinterpretation and to increase the trustworthiness of the study. Data triangulation was made between data collected in the interviews and in the FGDs. A member check was carried out with informal discussion with study participants to ensure that their perceptions were interpreted correctly. A strength of the study is that all authors have a public health perspective, have experience in qualitative research and have varied professional backgrounds viz. agriculture, environmental health, nursing and pharmacy. The different educational, geographical and cultural backgrounds of the authors, with extensive experience in their own field of research enhanced the conformability of the results. This is a qualitative study conducted in a tribal context based on focus group discussions and interviews. The findings of this study might however be of value for readers from a wide range of contexts with similar types of situations.

## 5. Conclusions

The perceptions and experiences of professional stakeholders suggest that water scarcity affects the hygiene and sanitation of the tribal people, and that it is the main hurdle for agricultural development. According to the participants, implementation of an integrated watershed management programme in tribal areas helps in increasing water availability and improvement in agriculture. This consequently will have a positive impact on economy, education and ultimately on public health. The causes of various health problems of tribal people in hilly areas are complex and are interlinked with water availability and socio-cultural factors. The study indicates that increase in availability and efficient use and management of water by implementation of IWMP can help to boost socio-economic development in tribal areas, resulting in positive health outcomes.

## Figures and Tables

**Table 1:** Background information of the individual interviewees.

Participants	Jurisdiction of Work	Number of Interviews	Age (Years)	Experience (Years)	Interview Length (Min)
Healthcare providers	District/Block/PHC	6	29–52	5–21	40–65
Development administrators	State/District/Block	8	35–65	1–35	47–119
Teachers	Village	3	30–60	4–25	40–63

Notes: PHC—Primary Health Center; Healthcare providers—physicians and drug dispensers; Development administrators—Development officers working in the field of child development and nutrition, and rural development in the government institutions as well as in NGOs.

**Table 2:** Background information of participants of the focus group discussions.

Participants	Sex	Occupational Background	Number of Participants	Age Range	FGD Length (Min)
ASHA/Anganwadi workers (Frontline healthcare providers)	Female	Healthcare	9	23–42	93
Local Leaders (Village level)	Male	Social/Political	7	25–40	95
Teachers	Male	Education	8	30–35	53
Village development officers	Male	Development Administration	8	32–50	45

Note: ASHA—Accredited Social Health Activists.

**Table 3:** Interview/focus group discussions (FGD) guide, showing the introductory questions.

Introductory Question
What is the situation of availability of drinking water for the community in the tribal villages?
What kinds of crops are grown by the tribal people in the villages? (For home consumption or sale)
How are hygiene and sanitary practices by villagers?
How is the situation of water related and other diseases in the villages?
What are the general health problems for the villagers?
Do you have any idea about integrated watershed management? (Explanation about watershed management if needed)
How can an integrated watershed management programme influence the health of the tribal people in this area?
Anything to add

**Table 4:** Theme—Possible impact of integrated watershed management on complex interlinked factors influencing health of tribal people.

Theme	Possible Impact of Integrated Watershed Management on Complex Interlinked Factors Influencing Health of Tribal People				
Categories	Impact of water scarcity on health issues	Effects of (lack of) education on health and utilization of healthcare facilities	Role of traditions on women’s and children’s health	Dependence of economy on agriculture	Efforts of the government to provide/improve education, employment and healthcare
Sub-categories	Water related diseases	Illiteracy	Traditions	Seasonal rain-fed agriculture	Schemes on education and nutrition
	Hygiene and sanitation practices	Misconceptions and superstitions	Hard work of women (Drudgery)	Forceful seasonal migration	Employment guarantee scheme
	Agricultural practices and food consumption	Alcoholism in men	Malnutrition		Health promotion schemes and activities
Example of codes	Stomach problem, Diarrhoal diseases, Hepatitis, Conjunctivitis, Malaria, Pneumonia. Distance of water source, Open defecation, Toilet use, Bathing, Washing of clothes, River, Hand washing, Tooth cleaning, Care of infants, Water contamination. Rain-fed agriculture, Seasonal crops, Crops mainly for self-consumption, No leafy vegetables.	Ignorance, No formal education, Awareness about health. Misconceptions, Traditions, Superstitions, Responsiveness Unemployment, Frustration, Alcohol consumption in men, Family health.	Early age marriages, Arranged marriages, Bathing of neonates in open with normal water, Alcohol consumption. Collection of water and firewood, Household work, Farm work, Hygiene maintenance. Under-weight children, Less consumption of oil, No milk consumption, Anemia in women.	Rainfed crops, Rice-nagali-bhagar, No irrigation facilities, Primitive agriculture. Seasonal migration, Unemployment, Source of earning cash.	Residential school, Free education, Mid- day meal, Anganwadi workers, ASHA workers. Development work by government, employment assurance. Cash incentives for institutional deliveries, Healthcare—mainly curative, Disinfection of drinking water, Lack of co-ordination
